# Editorial: Innovative Radiopharmaceuticals in Oncology and Neurology

**DOI:** 10.3389/fmed.2016.00074

**Published:** 2017-01-11

**Authors:** Jacques Barbet, Nicolas Arlicot, Marie-Hélène Gaugler, Michel Chérel, Denis Guilloteau, Françoise Kraeber-Bodéré

**Affiliations:** ^1^Centre Régional de Recherche en Cancérologie Nantes/Angers (CRCNA), UMR892 Inserm, Nantes, France; ^2^6299 CNRS, Nantes, France; ^3^Université de Nantes, Nantes, France; ^4^GIP Arronax, Saint-Herblain, France; ^5^UMR Inserm U930, Tours, France; ^6^Université François Rabelais, Tours, France; ^7^CHRU de Tours, Hôpital Bretonneau, Tours, France; ^8^Service de Médecine Nucléaire, Institut de Cancérologie de l’Ouest, Saint-Herblain, France; ^9^Service de Médecine Nucléaire, CHU de Nantes, Nantes, France

**Keywords:** radiopharmaceuticals, targeted radionuclide therapy, multimodality imaging, theranostic, PET imaging, pretargeted radioimmunotherapy

**Editorial on the Research Topic**

**Innovative Radiopharmaceuticals in Oncology and Neurology**

Personalized medicine is presented as the future of patient care. Nuclear Medicine will play a major role in the selection of patients for targeted therapies and in early therapy assessment with the investigation of phenotypes and functions using sensitive and specific SPECT and PET imaging techniques and theranostic approaches. Nuclear Medicine is already a key in the development of new therapies and targeted radionuclide therapy provides efficacious treatment modalities against cancer. An incredible amount of innovation is related to the use of a variety of radionuclides, new or improved multimodality imaging devices, and numbers of recently marketed radiopharmaceuticals in all medical domains and particularly cardiology, neurology, and oncology.

In this context, a network of laboratories and Nuclear Medicine departments, the IRON Laboratory of Excellence (Labex), has been set as part of the French “Investissements d’Avenir” program to help translating innovative radiopharmaceuticals into clinical testing by a multidisciplinary approach from the production of radionuclides using cyclotrons, to basic studies of targets and ligands, clinical trials, and assessment of the societal impacts of innovation in medicine in neurology and oncology. To foster communication between scientists, inside the Labex and with the international community, a series of highly specialized international “Nuclear Technologies for Health Symposia” has been organized annually, with the most recent edition, the third, held in Nantes, France, on March 10–11, 2015.

Plenary lectures given by distinguished speakers (Hank Kung, Philadelphia, PA, USA, Tom Bäck, Gothenburg, Sweden, Otto Boerman, Nijmegen, The Netherlands, Franck Bruchertseifer, Karlsruhe, Germany, and Claire Tabouret-Viaud, Geneva, Switzerland) addressed the state of the arts and new developments in the use of radioactivity and radiopharmaceuticals for multimodality imaging and therapy. The latest achievements in clinical nuclear medicine, science, and technology were discussed during interactive oral and poster sessions.

Selected talks were organized in four sessions: innovative tracers in neurology: from bench to bedside, multimodality imaging, theranostic in nuclear medicine, and targeted radionuclide therapy.

After the meeting, 12 papers were published in the Nuclear Medicine topic open by *Frontiers in Medicine*. These papers are representative of current research and development in Nuclear Medicine.

In a review article, Chatal et al. discussed the interest of ^82^Rb PET for heart diseases, especially in obese patients and in women with large breasts. A great advantage of ^82^Rb PET is its capacity to accurately measure myocardial blood flow and coronary reserve. ^82^Rb has a very short half-life (75 sec) and is obtained from an ^82^Sr/^82^Rb generator. A major limitation for this technology is the low worldwide production of ^82^Sr, but the situation is improving with new production facilities, including Arronax, becoming active, increased production yields, and approval of new generator/infuser systems.

Another radionuclide that attracts interest is ^64^Cu, a PET emitter with a longer half-life (12.7 h). ^64^Cu may be used to label peptides and antibodies. Another potential application is the mapping of tumor hypoxia using ^64^Cu-ATSM, as discussed by Colombié et al. Hypoxic cells are more resistant to chemotherapy and to external beam radiation therapy. Delivering increased radiation doses to tumor hypoxic areas delineated by PET imaging could improve response. However, there is some controversy about the actual cause of increased ^64^Cu-ATSM in tumor tissues. Corroyer-Dulmont et al. made a rather comprehensive review of the various methods that may be used to assess oxygenation/hypoxia, focusing on glioblastoma: other PET tracers such as [^18^F]Fluoromisonidazole ([^18^F]FMISO) as well as many other imaging approaches, including MRI. They conclude that PET imaging may be the most relevant tool to characterize hypoxia in glioblastoma.

PET imaging is also useful in neurodegenerative diseases with the new tracers of amyloid plaques and in stroke with tracers of neuroinflammation. The objective of the paper by Sérrière et al. was to evaluate the effects of a new agonist of α7R on striatal dopaminergic neurodegeneration and neuroinflammation in a rat model of Parkinson Disease by PET imaging with [^18^F]LBT-999 to quantify the striatal dopamine transporter. Thanks to the use of preclinical PET imaging, they could conclude that an α7R agonist may be beneficial for the treatment of Parkinson Disease.

In cancer diseases, [^18^F]FDG and [^18^F]fluorocholine are the most commonly used tracers in clinical practice. In a retrospective study, Rusu et al. evaluated the contribution of [^18^F]FDG PET to the clinical management and survival outcome of patients suspected of recurrent ovarian carcinoma. They showed that early diagnosis of recurrent ovarian cancer by PET had a significant impact on treatment planning and predicted patient outcome. In prostate cancer, Mathieu et al. reported interest of dynamic PET/CT image acquisition with [^18^F]fluorocholine to define pelvic lymph node or prostate pathological status.

Nuclear Medicine is also therapy. This has been known for many years, with the use of ^131^I in the treatment of thyroid cancer. Tracking other cancers remains a challenge, but progress is made. New radioactive therapeutic agents are designed to improve response and survival, while minimizing side-effects. Locoregional injection of radioactive microparticles is in use in hepatic cancers. Encapsulation of radionuclides in nanoparticles that could be injected systemically has been proposed as a way to increase the radioactive load and reduce exposure of normal tissues. Lepareur and coworkers described non-toxic nanoparticles, able to release their load in a controlled way. They prepared biodegradable and biocompatible pegylated nanoparticles loaded with a lipophilic ^99m^Tc-labeled tracer that may be labeled by ^188^Re for therapy (Lepareur et al.). Targeting similar nanoparticles is also explored by Rauscher et al., who develop pegylated liposomes labeled with ^111^In and ^125/131^I for pretargeted radioimmunotherapy, using a bispecific monoclonal antibody recognizing a tumor antigen and a small molecular weight tag attached to the surface of the liposomes. Immunospecific tumor targeting was demonstrated *in vitro* and *in vivo* in a mouse model.

With small radiolabeled molecules, clinical proofs of concept have been obtained for a variety of pretargeting methods in single photon imaging, PET imaging, and therapy. The key is the bispecific pretargeting agent and the optimization of the pretargeting protocol. Bodet-Milin et al. studied a new class of bispecific antibodies in a phase I radioimmunotherapy trial (http://ClinicalTrials.gov NCT01221675) to optimize pretargeting parameters. The best dosing parameters were a short pretargeting delay (24 h) and a high bispecific molar dose.

A promising avenue for cancer therapy is the use of alpha-particle emitters. Alpha particles have very short tracks (usually less than 100 µm) and a very high cytotoxic potential even against radioresistant tumor cells in hypoxic area. ^223^Ra, a bone targeting agent, has been recently approved for symptomatic metastatic prostate cancer therapy. Antibodies may be used to target other alpha-emitting radionuclides. Here, ^213^Bi has been targeted to multiple myeloma, in a syngeneic mouse model, by means of an anti-CD138 antibody. Fichou et al. demonstrated that ^213^Bi is much more effective than ^177^Lu. The higher efficacy in such disseminated diseases has been presented as the major interest and the best rationale for the use of alpha emitters in therapy, but this is one of the first direct comparisons. In the same model, Gorin et al. showed that autophagy is a prominent cell death mechanism with ^213^Bi. Derrien et al. confirmed the efficacy of locally delivered antibodies labeled with ^213^Bi in an ovarian cancer model and proposed combination with hyperthermic intraperitoneal chemotherapy in an attempt to treat advanced disease.

## Author Contributions

Substantial contributions to the conception or design of the work; or the acquisition, analysis, or interpretation of data for the work: JB, NA, M-HG, MC, DG, FK-B. Drafting the work or revising it critically for important intellectual content: JB, NA, M-HG, MC, DG, FK-B. Final approval of the version to be published: JB, NA, M-HG, MC, DG, FK-B. Agreement to be accountable for all aspects of the work in ensuring that questions related to the accuracy or integrity of any part of the work are appropriately investigated and resolved: JB, NA, M-HG, MC, DG, FK-B.

## Conflict of Interest Statement

The authors declare that the research was conducted in the absence of any commercial or financial relationships that could be construed as a potential conflict of interest. The handling Editor declared a shared affiliation, though no other collaboration, with the authors and states that the process nevertheless met the standards of a fair and objective review.

